# An Updated Meta-Analysis of DOACs vs. VKAs in Atrial Fibrillation Patients With Bioprosthetic Heart Valve

**DOI:** 10.3389/fcvm.2022.899906

**Published:** 2022-06-17

**Authors:** Yalin Cao, Yuxiang Zheng, Siyuan Li, Fuwei Liu, Zhengbiao Xue, Kang Yin, Jun Luo

**Affiliations:** ^1^Department of Cardiology, Guizhou Provincial People's Hospital, Guiyang, China; ^2^Second Clinical Medical College, Nanchang University, Nanchang, China; ^3^Department of Cardiology, The Affiliated Ganzhou Hospital of Nanchang University, Ganzhou, China; ^4^Department of Critical Care Medicine, the First Affiliated Hospital of Gannan Medical University, Ganzhou, China

**Keywords:** atrial fibrillation, anticoagulants, safety, effectiveness, meta-analysis

## Abstract

**Background:**

Current guidelines recommend the utilization of direct-acting oral anticoagulants (DOACs) in patients with non-valvular atrial fibrillation (AF). However, the optimal anticoagulation strategy for AF patients with bioprosthetic heart valves (BPHV) remains controversial. Therefore, we conducted this meta-analysis to explore the effect of DOACs versus vitamin K antagonists (VKAs) in this population.

**Methods:**

We systematically searched the PubMed and Embase databases until November 2021 for studies reporting the effect of DOACs versus VKAs in AF patients with BPHV. Adjusted risk ratios (RRs) and 95% confidence intervals (CIs) were pooled using the random-effects model with an inverse variance method.

**Results:**

We selected four randomized clinical trials and seven observational studies (2236 DOAC- and 6403 VKAs-users). Regarding the effectiveness outcomes, there were no significant differences between DOACs and VKAs in stroke or systemic embolism (RR = 0.74, 95%CI: 0.50–1.08), ischemic stroke (RR = 1.08, 95%CI: 0.76–1.55), all-cause death (RR = 0.98, 95%CI: 0.86–1.12), and cardiovascular death (RR = 0.85, 95%CI: 0.40–1.80). In terms of the safety outcomes, DOACs was associated with lower risks of major bleeding (RR = 0.70, 95%CI: 0.59–0.82) and intracranial bleeding (RR = 0.42, 95%CI: 0.26–0.70), but the risks of any bleeding (RR = 0.85, 95%CI: 0.65–1.13) and gastrointestinal bleeding (RR = 0.92, 95%CI: 0.73–1.17) are not significantly different when compared with VKAs. The subgroup analysis with follow-up as a covariate revealed that the DOACs had lower risks of SSE (RR = 0.59, 95%CI: 0.37–0.94) and major bleeding (RR = 0.69, 95%CI: 0.58–0.81) in patients with a mean follow-up of more than 24 months, but no statistical differences were found in patients with the follow-up less than 24 months (SSE: RR = 1.10, 95%CI: 0.92–1.32; major bleeding: RR = 0.91, 95%CI: 0.42–2.01).

**Conclusions:**

In AF with BPHV, patients on DOACs experienced a reduced risk of major bleeding and intracranial bleeding compared with VKAs, while the risks of stroke, cardiovascular death, and all-cause mortality were similar.

## Introduction

Atrial fibrillation (AF) is the most common arrhythmia among adults, affecting an estimated 1.2 million people in the UK (1). Characterized by rhythm irregularity, AF patients are prone to forming thrombi in the left atrium/left atrial appendage due to stasis of blood and are at risk of thromboembolic events (2). Moreover, AF may be of valvular etiology or non-valvular. The presence of valvular disease further complicates the course of AF and tends to increase morbidity and mortality. Consequently, anticoagulation therapy becomes an indispensable part of preventing thromboembolic events for patients with AF and valvular heart disease (VHD).

Direct-acting oral anticoagulants (DOACs) have been considered the first-line choice for non-valvular AF patients (3). However, when it comes to patients with bioprosthetic heart valves (BPHV), the use of DOACs is contraindicated to a large extent, and warfarin is the only permitted oral anticoagulant (4). Both the American College of Cardiology (5) and major Japanese guidelines (6–8) do not endorse the use of DOACs after bioprosthetic valve replacement (BVR). Conversely, the European Society of Cardiology (9) and the European Heart Rhythm Association (10) states that DOACs should be considered in patients with AF and bioprosthetic heart valve (BPHV), but no earlier than 3 months after bioprosthetic aortic valve replacement. Nonetheless, concerning the lower PT-INR settings in Asia, the racial differences in thromboembolism or bleeding prevalence between Asian and western patients (11) and the lack of robust evidence, the results of Asian patients should not be simply generalized to the western population, and more updated researches for a clear consensus guideline are integral.

With the ever-increasing number of observational studies supporting strong evidence to the issue, we conducted the meta-analysis to better understand the effectiveness and safety of DOACs in AF patients with BPHV. It incorporated a larger patient population and considered more factors, identifing the optimal antithrombotic strategies in real-world clinical practice.

## Methods

Throughout this meta-analysis, the Preferred Reporting Items for Systematic Reviews and Meta-Analysis (PRISMA) guidelines for all stages of the design and implementation were followed (12). There was no need for ethical approval as only published studies were included.

### Searching Strategy

We systematically searched the PubMed and Embase databases from inception to November 2021 with the following search terms: (1) *atrial fibrillation*, (2) *edoxaban* OR *dabigatran* OR *rivaroxaban* OR *apixaban* OR *non-vitamin K oral anticoagulants* OR *direct oral anticoagulants* OR *novel oral anticoagulants OR DOAC* OR *NOAC*, (3) *biologic valve* OR *bioprosthetic valve* OR *biological valve* OR *bioprosthesis*, (4) *warfarin* OR *vitamin K antagonists* OR *VKA* OR *coumadin* OR *dicoumarol* OR *acenocoumarol*. The detailed searching strategies are shown in Supplementary Table 1. No language restrictions were applied in this meta-analysis.

### Eligibility Criteria

We included the randomized controlled trials (RCTs), *post-hoc* analyses of RCTs and observational cohort studies focusing on the effectiveness and/or safety of DOACs (dabigatran, rivaroxaban, apixaban, or edoxaban) compared with VKAs in AF patients with BPHV. We included the simultaneously reported outcomes in at least two included articles. Our effectiveness outcomes included stroke or systemic embolism (SSE), ischemic stroke, all-cause death, and cardiovascular death, whereas the safety outcomes included major bleeding, gastrointestinal bleeding, intracranial bleeding, and any bleeding. Thereinto, the primary effectiveness and safety outcomes were SSE and major bleeding, respectively. The studied outcomes and their definitions were chosen according to the originally included studies and the definitions were shown in Supplementary Table 2. Studies would be excluded if they had no sufficient data (e.g., comments, case reports, reviews, editorials, letters) or did not report the quantitative effect estimate. Studies involving mechanical heart valves, rheumatic valvular disease, and overlapping data were also excluded. In addition, studies that did not report stroke, systemic embolism, and major bleeding outcomes separately were also excluded.

### Study Selection and Data Extraction

Two independent researchers first screened the titles and abstracts of the retrieved records and then viewed the full-texts of the potential studies for the second screening. Disagreements were resolved through discussion with each other or with the third reviewer. Data were collected as follows: the first author and publication year, study design, data source, the study characteristics, type of DOACs, number of DOAC- or VKA-users, length of follow-up, effectiveness, and safety outcomes.

### Quality Assessment

The Cochrane risk of bias assessment tool evaluated the methodological quality of RCTs and *post-hoc* analysis of RCTs. The Newcastle-Ottawa Scale (NOS) tool was applied to assess the study quality for observational cohorts. The NOS tool included three major sections as follows: the selection of cohorts (0-4 points), the comparability of cohorts (0-2 points), and the assessment of the outcome (0-3 points). We regarded the NOS score of ≥6 points as a moderate-to-high quality, while a NOS score of <6 points as a low-quality (13).

### Statistical Analysis

The statistical heterogeneity across the included studies was assessed using the *P*-value of the Cochrane Q-test and the *I*^2^ value. The *I*^2^ test was interpreted as follows: 0–40% might not be important, 30–60% may indicate moderate heterogeneity, 50–90% indicates substantial heterogeneity and over 75% indicates considerable heterogeneity.First, the number of participants and events were compiled in each group, and their corresponding crude rates of effectiveness and safety outcomes were worked out, represented by odds ratios (ORs) and 95%CIs. Second, we reckoned the relevant outcomes using the adjusted RRs and converted the adjusted RRs and 95%CI to the natural logarithms and standard errors. All the comparison results were pooled by a random-effects model using an inverse variance method. The publication bias was evaluated for the effect estimates based on the funnel plots.

We used the Review Manager version 5.4 software (the Cochrane Collaboration 2014, Nordic Cochrane Centre Copenhagen, Denmark; https://community.cochrane.org/) to perform the meta-analysis. The statistical significance threshold was set at a *P*-value of < 0.05.

## Results

### Study Selection

**Figure 1:**
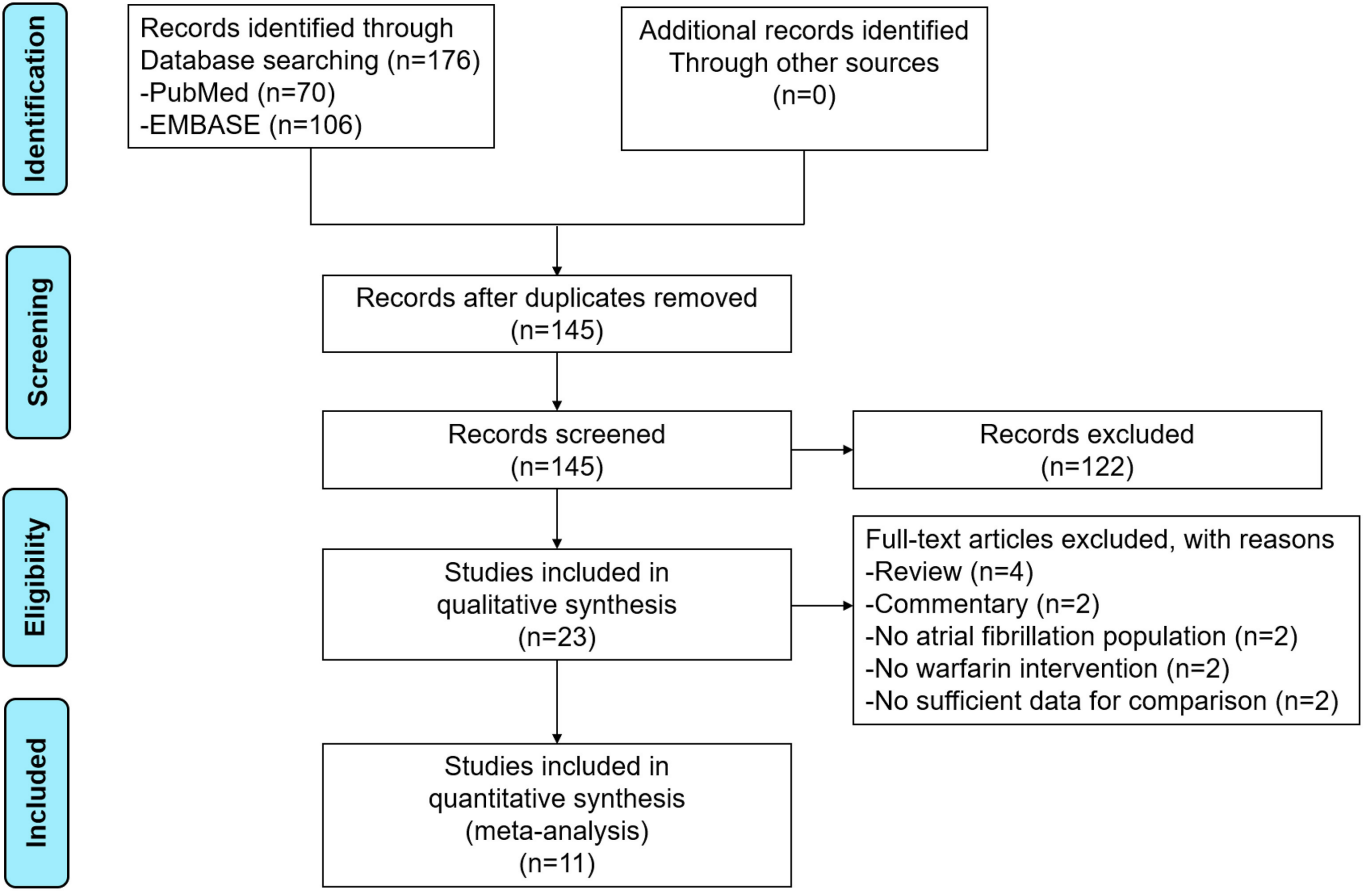
The process of the literature retrieval of this meta-analysis.

The process of the literature retrieval is presented in Figure 1. Through searching the electronic searches in the PubMed and EMBASE databases, our initial search yielded 176 articles. After the records screening, we selected 23 relevant articles. Subsequently, the full-text screening led to the exclusion of 12 articles based on the predefined criteria. Finally, a total of 11 studies [two *post-hoc* analyses of RCTs (14, 15), 2 RCTs (16, 17), and seven observational studies (18–24)] were included in our meta-analysis. The baseline characteristics of the included studies are illustrated in Table 1. All 11 included studies were published from 2016 to 2021, with the sample sizes ranging from 27 to 2,672. Participants in these studies ranged from 37 to 88.9 years old. For the quality assessment, both of the two RCTs and two *post-hoc* analyses of RCTs had a low risk of bias (Supplementary Table 3), whereas the seven observational studies had a moderate-to-high quality with a NOS of ≥6 points (Supplementary Table 4).

**Table 1:** Baseline characteristics of the included studies in this meta-analysis.

**Study(author)/Study name**	**Region**	**Study design**	**Participants (N)**	**Age (years)**	**HAS-BLED**	**CHA2DS2-VASc**	**Male ratio(%)**	**DOACs regimen**	**Follow-up (months)**	**Bioprosthetic valve types**
Carnicelli et al. (14)	Multi-center (America, Europe, Asia–Pacific region and South Africa)	*Post-hoc* analysis of ENGAGE AF-TIMI48	191	75.0	2.7	3.0	63.4	EDO	33.6	Mitral or aortic
Durães et al. (16) DAWA Pilot Study	Brazil	RCT	27	44.6	NA	NA	37.0	DA	3.0	Mitral and/or aortic
Guimarães et al. (15)	Multi-center (America, Europe and Asia Pacific)	*Post-hoc* analysis of ARISTOTLE	156	72.9	2.0	2.0	60.9	API	21.6	Mitral and/or aortic valve replacement or native valve repair
Guimarães et al. (17) RIVER	Brazil	RCT	1,005	59.3	1.6	2.6	39.6	RIV	39	Mitral valve
Russo et al. (23)	5 cardiologic centers in Italy	Observational study	260	65.9	1.2	3.1	56.0	EDO, DA, API, RIV	26.8	Mitral or aortic
Duan et al. (18)	America	Observational study	2,672	NA	NA	NA	NA	DA, API, RIV	34.8	Mitral and/or aortic
Mannacio et al. (21)	Italy	Observational study	642	NA	NA	NA	NA	DA, RIV, API, EDO	38.4	Aortic valve
Myllykangas et al. (22)	Finnish	Observational study	2,245*	75.4	NA	NA	57.3	DA, RIV, API, EDO	36.0	Aortic valve
Strange et al. (24)	Denmark	Observational study	397	78.6	2.6	3.6	NA	RIV, API	24.0	Mitral and/or aortic
Izumi et al. (8)	Japan	Observational study	214	76.8	3.6 ± 1.2	4.0	46.7	NA	46.0	Mitral and/or aortic
Izumi et al. (19)	Japan	Observational study (Data from BPV-AF Registry)	752	81.3	2.5	4.3	44.7	NA	12.0	Mitral and/or aortic

*RCTs, randomized controlled trials; DOACs, direct-acting oral anticoagulants; DA, dabigatran; RIV, rivaroxaban; API, apixaban; EDO, edoxaban; HAS-BLED, Hypertension, Abnormal Renal/Liver Function, Stroke, Bleeding History or Predisposition, Labile International Normalized Ratio, Elderly, Drugs/Alcohol; CHA2DS2-VASc, congestive heart failure, hypertension, age ≥75 y (doubled), diabetes mellitus, stroke (doubled)-vascular disease, age 65–74 and sex category (female)*.

^*^ Patient in this study was changed into another group if the medication was changed, so 2,158 patients were in warfarin group and 168 patients were in DOACs group.

### Crude Event Rate Between DOACs vs. VKAs

Ten included studies reported the crude rates of effectiveness or safety outcomes between DOACs vs. VKAs (14–17, 19–25). For the effectiveness outcomes shown in Supplementary Figure 1, compared with VKAs, no statistically difference was represented in SSE (OR = 0.70, 95%CI: 0.47–1.02), ischemic stroke (OR = 0.71, 95%CI: 0.33–1.55), all-cause death (OR = 0.81, 95%CI: 0.47–1.37) and cardiovascular death (OR = 0.89, 95%CI: 0.47–1.67).

The safety outcomes of DOACs vs. VKAs are presented in Supplementary Figure 2. The pooled analysis demonstrated that DOAC-users had lower event rates of major bleeding (OR = 0.60, 95%CI: 0.42–0.84) compared with VKA-users, whereas the rates of any bleeding (OR=0.83, 95%CI: 0.57–1.20), and intracranial bleeding (OR = 0.84, 95%CI: 0.26–2.66) between the two studied groups were similar.

### Adjusted Data of Outcomes Between DOACs vs. VKAs

**Figure 2:**
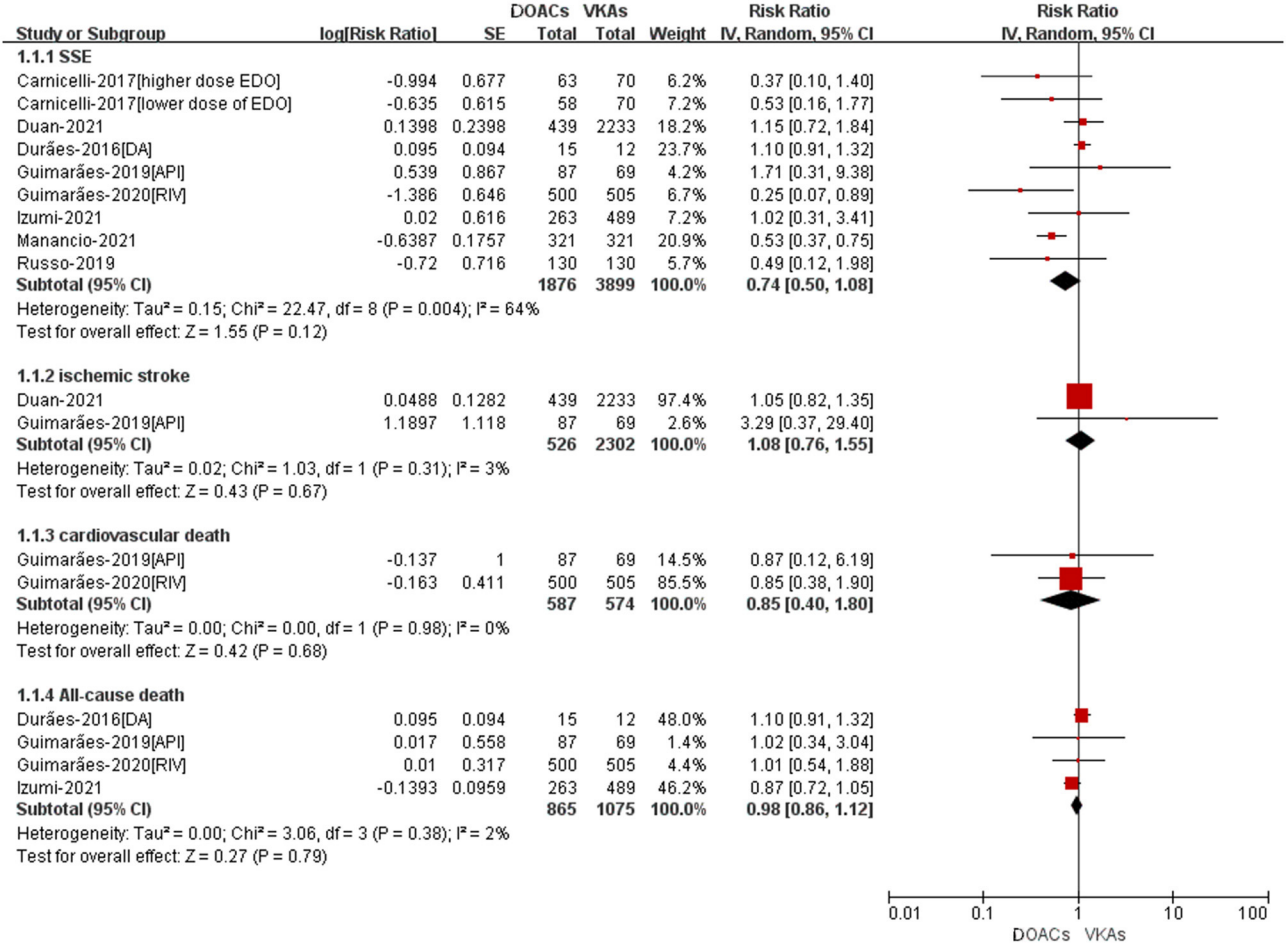
Adjusted effectiveness data of DOACs compared with VKAs among AF patients with BPHV. SSE, Stroke or systemic embolism; RCTs, randomized controlled trials; DOACs, direct-acting oral anticoagulants; CI, confidence interval; VKAs, vitamin K antagonists.

A total of eight studies reported the adjusted data of efffectiveness or safety outcomes between DOACs vs. VKAs (14–19, 21, 23). As shown in Figure 2, for the effectiveness outcomes, there was no significant differences between DOACand VKA groups in SSE (RR = 0.74, 95%CI: 0.50–1.08), ischemic stroke (RR = 1.08, 95%CI: 0.76–1.55), all-cause death (RR = 0.98, 95%CI: 0.86–1.12), and cardiovascular death (RR = 0.85, 95%CI: 0.40–1.80).

**Figure 3:**
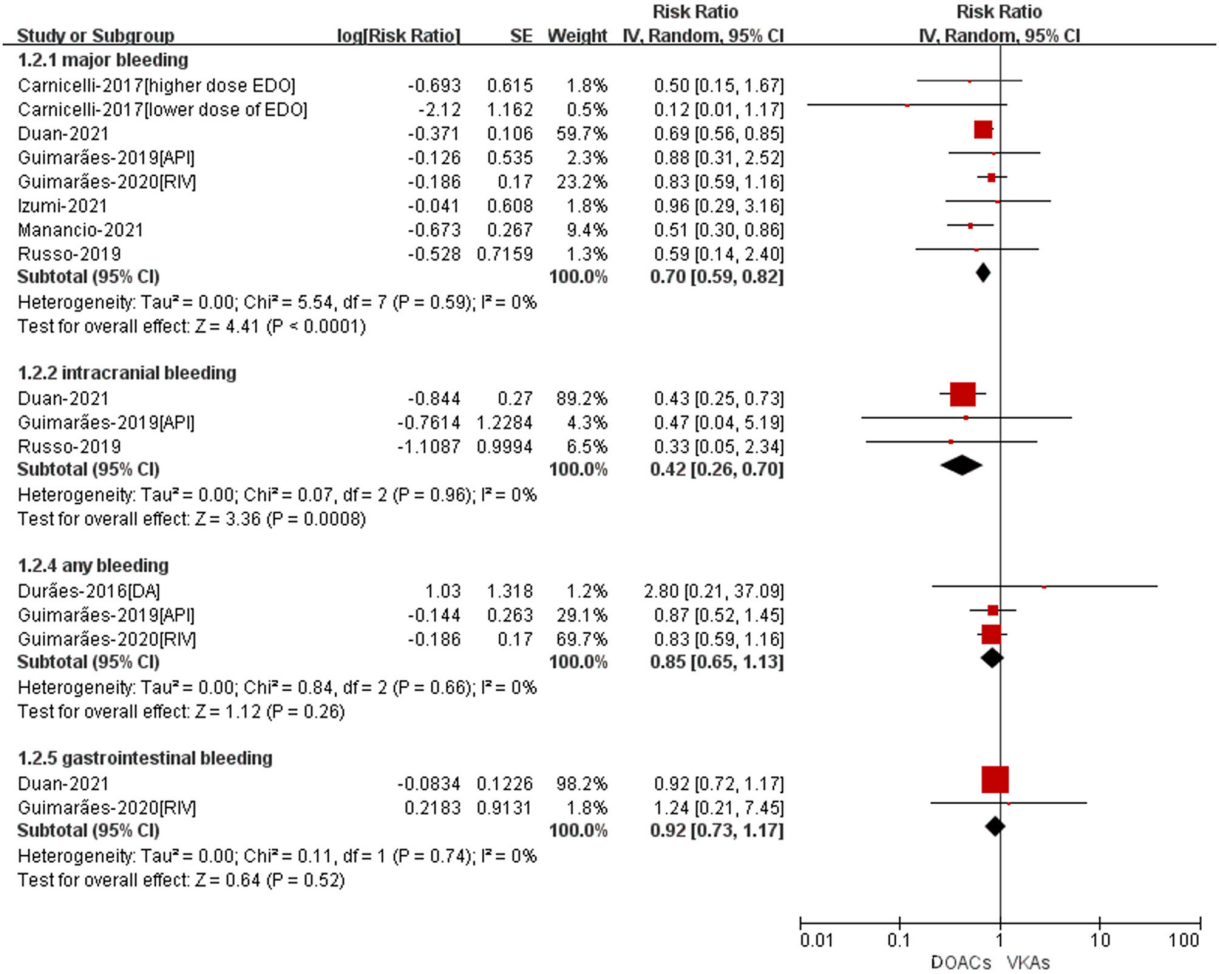
Adjusted safety data of DOACs compared with VKAs among AF patients with BPHV. RCTs, randomized controlled trials; DOACs, direct-acting oral anticoagulants; CI, confidence interval; VKAs, vitamin K antagonists.

The safety outcomes were shown in Figure 3. Compared with VKA-users, the use of DOACs was significant associated with reduced risks of major bleeding (RR = 0.70, 95%CI: 0.59–0.82) and intracranial bleeding (RR = 0.42, 95%CI: 0.26–0.70). There was no statistically differences in any bleeding (RR = 0.85, 95%CI: 0.65–1.13) and gastrointestinal bleeding (RR = 0.92, 95%CI: 0.73–1.17) between patients treated with DOACs compared to patients treated with VKAs.

### Subgroup Analysis

**Figure 4:**
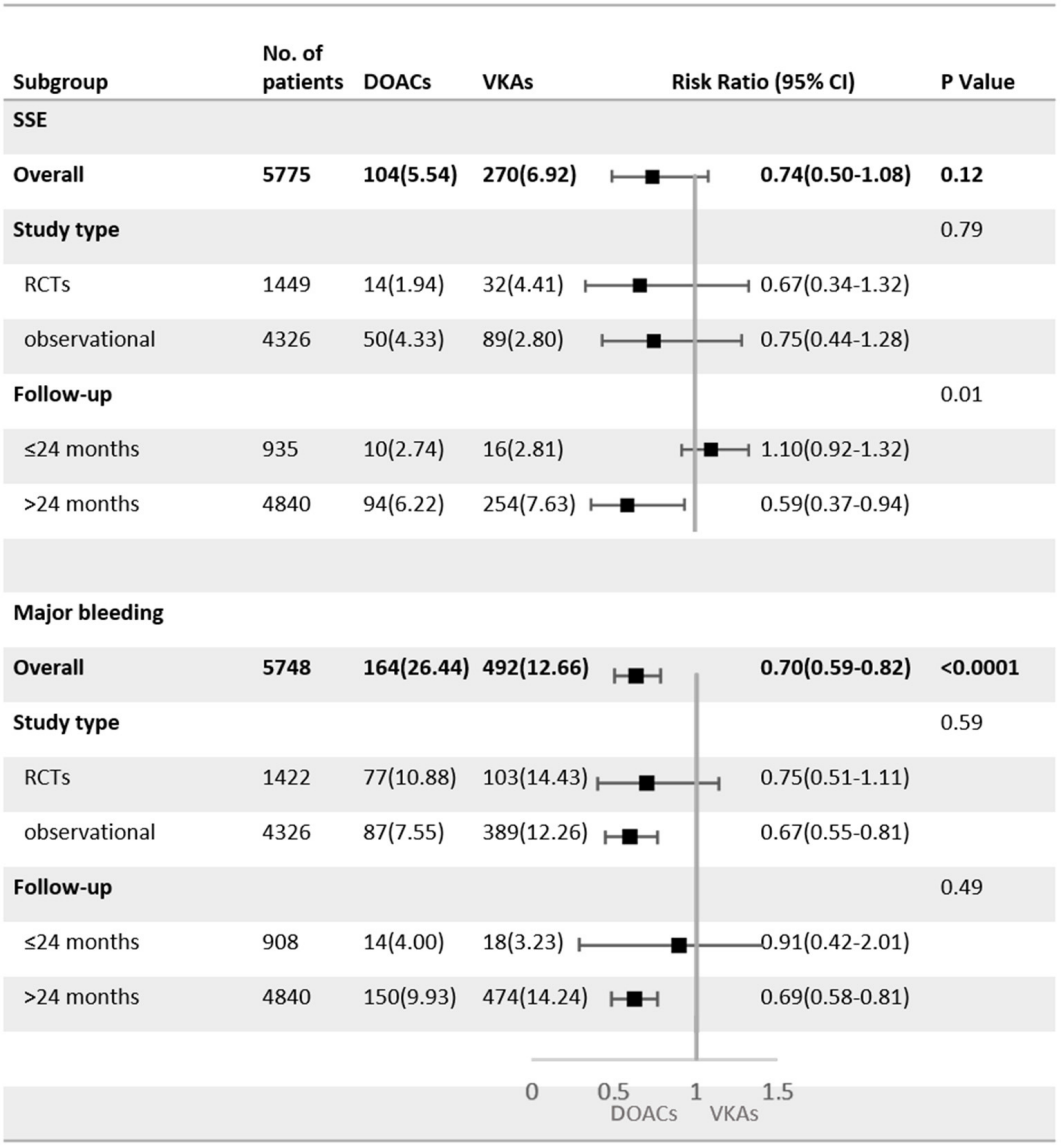
Subgroup analysis of adjusted efficacy and safety data of DOACs compared with VKAs among AF patients with BPHV. SSE, Stroke or systemic embolism; RCTs, randomized controlled trials; DOACs, direct-acting oral anticoagulants; CI, confidence interval; VKAs, vitamin K antagonists.

As shown in Figure 4, SSE and major bleeding outcomes were consistent between the observational studies and RCTs (P for interaction = 0.79 for SSE; P for interaction = 0.59 for major bleeding). For patients treated with DOACs compared with VKAs, the risk of major bleeding did not show a significant difference between groups in RCTs (RR = 0.75, 95%CI: 0.51–1.11), but was statistically different in observational studies (RR = 0.67, 95%CI: 0.55–0.81).

The subgroup analysis with follow-up as a covariate revealed that the DOACs had lower risks of SSE (RR = 0.59, 95%CI: 0.37–0.94) and major bleeding (RR = 0.69, 95%CI: 0.58–0.81) in patients with a mean follow-up of more than 24 months, but no statistical differences were found in patients with the follow-up <24 months (SSE: RR = 1.10, 95%CI: 0.92–1.32; major bleeding: RR = 0.91, 95%CI: 0.42–2.01).

### Publication Bias

As shown in Supplementary Figures 3, 4, no obvious publication biases were observed when assessed by using the funnel plots. Also, it was noted that the publication bias should not be evaluated when the included studies of the outcome were fewer than 10.

## Discussion

Our systematic analysis among patients with AF and BPHV indicated the following results: (1) In comparison with VKAs, DOACs were non-inferior regarding the outcomes of SSE, ischemic stroke, all-cause death and cardiovascular death. (2) As a class, DOACs were connected with decreased risk of major bleeding and intracranial bleeding as compared with VKAs. (3) DOACs were non-inferior regarding the outcomes of gastrointestinal bleeding and any bleeding.

Considering that AF patients with BPHV require long-term anticoagulation and this patient population has grown by leaps and bounds (26), finding the optimal anticoagulant treatment is critical. On the one hand, an increasing number of elderly patients undergoing BVR are affected by high cardiovascular risk factors such as hypertension, diabetes, and stroke history. They are not only susceptible to thromboembolism events, but also to bleeding events during anticoagulation therapy. On the other hand, patients with AF have an inherent risk of thromboembolic disease, which is further complicated when AF is accompanied by with BPHV (27). It has been reported that the leaflet surface is prone to microthrombi and the fabric of the sewing ring remains exposed without neointimal coverage in the 3 weeks after BVR (28, 29), all of which contribute to the higher incidence of thrombosis.

VKAs have been widely used to prevent SSE in large populations and exert an effective influence on thromboembolism, but they have a narrow therapeutic range that requires close monitoring and dose or diet adjustments in clinical practice. By the way, DOACs are still more effective and safer than VKAs in AF patients during the optimal time period in the therapeutic range. Up to date, questions remain about the most effective treatment for AF patients with BPHV. In the Effective Anticoagulation with Factor Xa Next Generation in Atrial Fibrillation–Thrombolysis in Myocardial Infarction 48 (ENGAGE AF–TIMI 48) trial (14), a subgroup analysis of 131 patients with bioprosthetic mitral valves demonstrated a significantly lower rate of major bleeding in patients recieving lower-dose (30 mg) edoxaban, compared with the warfarin group. Likewise, several observational studies have reported that the use of DOACs in AF patients with BPHV appearsto be safe and effective in the treatment of thromboembolic events (30, 31). Growing evidence suggeststhat DOACs may represent a valid therapy for AF patients with BPHV. However, the current RCT conducted by Guimarães et.al stated that rivaroxaban was non-inferior to warfarin for the mean time until the occurrence of death, major cardiovascular events, or major bleeding at 12 months (17). Prior trials have shown that rivaroxaban was not inferior to warfarin for the prevention of SSE in ROCKET AF (32). The ARISTORLE trial also showed no significant differences between apixaban and warfarin for major bleeding or SSE for patients with BPHV and AF (15). Therefore, the large uncertainty of thromboembolic risk, concerns about bleeding complications as well as the paucity of evidence-based data limited the use of DOACs in AF patients with BPHV.

Recently, the effectiveness and safety of DOACs compared with VKAs in AF patients with BPHV have been explored in several studies (33–37) as shown in Supplementary Table 5. A prior systematic review by Kheiri et. al supported that SSE, mortality, and safety profiles of DOACs in AF patients with BPHV appeared to be similar to those in warfarin treatment (35). Cardoso et al. also performed a meta-analysis by including 2 *post-hoc* analyses of RCTs and two RCTs, suggesting that DOACs were associated with a reduced incidence of SSE and major bleeding as compared with warfarin in AF patients with BPHV (34). In addition to RCTs, the meta-analyses by Adhikari et al., Lacy et al., and Yokoyama et al. included a different number of observational studies (33, 36, 37). To our knowledge, this study is the largest to assess evidence in separate meta-analyses of RCTs (*n* = 4) and observational studies (*n* = 7) for DOACs compared with VKAs in AF patients with BPHV.

Our findings were largely consistent with the previous meta-analyses of RCTs and the recent meta-analyses, including a small number of observational studies. In addition, our screening criteria for patients undergoing BVR were more stringent, including only traditional biological valves. Notably, it is discovered that the results from the RCTs using DOACs for AF patients with BPHV did not find a decreased risk of major bleeding compared with VKAs as seen in the observational studies. Possible explanations include different follow-up durations, diverse definitions of outcomes, different assessment tools, the interaction between former treatment and DOACs (e.g., catheter ablation), and other unmeasured confounders. For instance, the definition of major bleeding varied across the observational studies and the CHA2DS2-VASc was not adopted in all of the observational studies (*n* = 5) for predicting the risk of stroke, which may disturb the population-based risk stratification and thus lead to inconsistency. An interesting thing that we analyzed in the subgroup analysis was that the DOACs had lower risks of SSE and major bleeding in patients with a mean follow-up of more than 24 months, which may promote the long-term use of DOACs in AF patients with BPHV.

Meanwhile, although the observational studies in our meta-analyses represented a wider range of age, CHA2DS2-VASc score, and follow-up duration than the RCTs, the overall results showed that the DOACs are comparable or superior to VKAs in terms of effectiveness and safety, providing evidence for the use of DOACs in a broader patient population than RCTs. In addition, we assessed crude event rates and adjusted data of outcomes between DOACs vs. VKAs in AF patients with BPHV. Above all, in comparison to VKAs, DOACs appeared to significantly reduce major bleeding and intracranial bleeding but showed comparable rates of SSE, ischemic stroke, all-cause death, cardiovascular death, gastrointestinal bleeding, and any bleeding.

### Limitations of Study

Shortcomings still exist in our meta-analysis. A significant limitation of our study was the lack of trials with head-to-head comparisons between different DOACagents and all the comparisons made between them were indirect. Second, although we have demonstrated that DOACs reduced the incidence of major bleeding and intracranial bleeding and performed similarly in other outcomes in patients with AF and BPHV, the credibility of the research is still poor as we included seven observational studies and two subgroup analyses of RCTs. Third, it should have been more specific about the accurate adjustment of DOACdose and the position of the bioprosthetic valve, so well-adjusted and robust population-based data are pursued further clinical application.

## Conclusion

Available data suggested that DOACs appear to reduce the risks of major bleeding and intracranial bleeding without raising the risk of SSE compared with VKAs among patients with AF and BPHV.

## Data Availability Statement

The original contributions presented in the study are included in the article/Supplementary Material, further inquiries can be directed to the corresponding author/s.

## Author Contributions

All authors listed have made a substantial, direct, and intellectual contribution to the work and approved it for publication.

## Funding

The Clinical Research Center Project of Department of Science & Technology of Guizhou Province [grant no. (2017) 5405].

## Conflict of Interest

The authors declare that the research was conducted in the absence of any commercial or financial relationships that could be construed as a potential conflict of interest.

## Publisher's Note

All claims expressed in this article are solely those of the authors and do not necessarily represent those of their affiliated organizations, or those of the publisher, the editors and the reviewers. Any product that may be evaluated in this article, or claim that may be made by its manufacturer, is not guaranteed or endorsed by the publisher.
