# Supplementary material for: An Updated Meta-Analysis of DOACs vs. VKAs in Atrial Fibrillation Patients With Bioprosthetic Heart Valve
Source: Front Cardiovasc Med. 2022 Jun 17;9:899906. doi: 10.3389/fcvm.2022.899906 (PMC9248967; doi:10.3389/fcvm.2022.899906)
Supplement: Supplementary file 1 [file Data_Sheet_1.PDF]

Supplementary Table 1. The search strategies until November 2021

	Search terms	Pubmed	Embase
#1	atrial fibrillation	93989	196329
#2	dabigatran	6068	18859
#3	rivaroxaban	6959	22789
#4	apixaban	4538	16453
#5	edoxaban	1870	6396
#6	Non-vitamin K oral anticoagulants	1510	607
#7	Direct oral anticoagulants	7521	2247
#8	Novel oral anticoagulants	2725	2092
#9	DOAC	2051	4303
#10	NOAC	1662	3771
#11	#2 OR #3 OR #4 OR #5 OR #6 OR #7 OR #8 OR #9 OR #10	18344	40315
#12	bioprosthetic valve	4127	2119
#13	biologic valve	14541	108
#14	biological valve	14551	497
#15	bioprosthesis	13901	14911
#16	#12 OR #13 OR #14 OR #15	28241	16024
#17	Vitamin K antagonists	9486	8027
	VKA	2233	4875
#18	warfarin	32067	103795
#19	dicoumarol	2804	4170
#20	acenocoumarol	1755	6918
#21	coumadin	32555	5016
#22	#17 OR #18 OR #19 OR #20 OR #21	43185	118023
#23	#1 AND #11 AND #16 AND #22	70	106

Supplementary Table 2. definition of the outcomes in the included studies

Study(author-year)	Definition of results
Carnicelli et al. -2017	Major bleeding: ISTH; All primary and secondary end points were adjudicated by independent blinded central event adjudication committee members.
Durães et al. -2016	Major bleeding: ISTH; The primary endpoint was the detection of intracardiac thrombus in TEE at the end of follow-up (90 days).
Guimarães et al.-2019	Study outcomes were adjudicated by an independent committee blinded to study drug assignment.
Guimarães et al.-2020	Major bleeding: TIMI and BARC
Russo et al.-2019	Major bleeding: ISTH; Both ischemic stroke and TIA were diagnosed by a neurologist.
Duan et al.-2021	Outcomes were identified using ICD-9 and ICD-10 codes in the primary discharge diagnosis position for inpatient hospitalizations.
Mannacio et al.-2021	Endpoints were adjudicated by experienced cardiologists or cardiac surgeons.
Monna et al.-2021	Intracranial bleeding was defined with ICD-10 codes I61 and I62; The gastrointestinal bleeding was defined with ICD-10 codes I85, K22.6, K25-29, K62.5, K66.1 and K92; Stroke was defined with the ICD-10 codes I63 and I64.
Strange et al.-2020	The inclusion data were identified using International Classification of Disease (ICD)-10 codes
Izumi et al.-2020	Major bleeding: ISTH
Izumi et al.-2021	Major bleeding: ISTH

Abbreviations: ISTH= International Society on Thrombosis and Haemostasis; TIMI= Thrombolysis in Myocardial In-farction; BARC= Bleeding Academic Research Consortium; ICD= International Classification of Disease;

Supplementary Table 3. Risk of bias assessment for RCTs and post-hoc analyses of RCTs

	Random sequence generation (selection bias)	Allocation concealment (selection bias)	Blinding of participants and personnel (performance bias)	Blinding of outcome assessment (detection bias)	Incomplete outcome data (attrition bias)	Selective reporting (reporting bias)	Other bias	Total*
Duraes-2016	Low risk	Low risk	Low risk	Low risk	Low risk	Low risk	Unclear risk	Low
Guimaraes-2020	Low risk	Low risk	Low risk	Low risk	Low risk	Low risk	Low risk	Low
Guimaraes-2019	Low risk	Low risk	Low risk	Low risk	Low risk	Low risk	Low risk	Low
Carnicelli-2017	Low risk	Low risk	Low risk	Low risk	Unclear risk	Low risk	Low risk	Low

*We defined as “low risk” when 3 out of 5 biases were “low”.

Supplementary Table 4. Quality assessment for the included post-hoc analyses of observational studies

Included studies	Selection (0-4 points)				Comparability (0-2 points)		Outcome (0-3 points)			Total poitns*
	Representativeness of Exposed Cohort	Selection of Non-Exposed Cohort	Ascertainment of Exposure	Demonstration That Outcome of Interest Was Not Present at Start of Study	Adjust for the important Risk factors	Adjust for other risk factors	Assessment of outcome	Follow-up length	Loss to follow-up rate	
Strange-2020	*	*	*		*	*	*	*	*	8
Biase-2021	*	*	*		*	*	*	*	*	8
Russo-2019	*	*	*		*	*	*	*	*	8
Duan L-2021	*		*			*	*	*	*	6
Mannacio-2021	*	*	*		*	*	*		*	7
Monna E-2021		*	*		*	*		*	*	6
Izumi-2020	*	*	*		*		*	*	*	7
Izumi-2021	*	*	*		*		*	*	*	7

*The Newcastle-Ottawa Scale (NOS) items, with a total score of 9 points, were used to evaluate the quality of the post-hoc analyses of RCTs which involve the selection of cohorts (0-4 points), the comparability of cohorts (0-2 points), and the assessment of the outcome (0-3 points)

Supplementary Table 5. Comparison with other relevant meta-analyses

Study	Number of articles included	Participants (N)	Measure of outcome	Subgroup analysis	Analysis method	Results (DOAC vs. warfarin)
Adnikari et al.	2 post-hoc analyses of RCTs, 2 RCTs and 1 observational study	1776	SSE, major bleeding, ICH all-cause mortality	/	HRs and 95%CIs	Non-significant reduction in SSE, lower incidence of major bleeding
Yokoyama et al.	2 post-hoc analyses of RCTs, 2RCTs and 6 observational studies (patients with TAVR are included, such as Seeger J. et.al, Butt et. al, and Kawashima et.al)	6405	All-cause mortality, SSE Major bleeding	All the outcomes were consistent between the RCTs and the observational studies	HRs and 95%CIs	reduced incidence of major bleeding
Ruzieh et al	2 RCTs, 2 post-hoc analysis of RCT and 4 observational studies (patients with TAVR are included, such as Butt et.al)	5300	all-cause mortality, cardiovascular death, SSE, intracranial bleeding, major bleeding, minor bleeding	/	RRs and 95%CIs	No significant difference in all outcomes
Lacy et al.	2 post-hoc analyses of RCTs, 2RCTs and 2 observational studies (patients with TAVR are included, such as Seeger J. et.al)	1911	Stroke, major bleeding, mortality	Age(age>60/ age<60)	RRs and 95%CIs	reduced incidence of stroke and major bleeding
Kheiri et al.	2 post-hoc analyses of RCTs and 2RCTs	1379	SSE, all-cause death, any bleeding, major bleeding	/	HRs and 95%CIs	Noninferior in the incidence of stroke, SSE, bleeding and mortality
Cardoso et al.	2 post-hoc analyses of RCTs and 2RCTs	1379	SSE, ischemic stroke, hemorrhagic stroke, cardiovascular death, all-cause mortality, major bleeding	/	ORs and 95%CIs	reduced incidence of SSE and major bleeding

Abbreviations： SSE = Stroke or systemic embolism; RCTs = randomized controlled trials; DOACs= direct-acting oral anticoagulants; TAVR= transfemoral aortic valve replacement; OR=odds ratio; HRs=hazard ratios; CIs=confidence intervals

Study or Subgroup	DOACs Events	DOACs Total	VKAs Events	VKAs Total	Weight	Odds Ratio M-H, Random, 95% CI
**2.1.1 SSE**						
Guimarães-2020[RIV]	3	500	13	505	7.6%	0.23 [0.06, 0.81]
Durães-2016[DA]	0	15	1	12	1.3%	0.25 [0.01, 6.64]
Carnicelli-2017[higher dose EDO]	3	63	8	70	6.6%	0.39 [0.10, 1.53]
Manancio-2021	42	321	70	321	27.8%	0.54 [0.36, 0.82]
Carnicelli-2017[lower dose of EDO]	4	58	8	70	7.7%	0.57 [0.16, 2.01]
Russo-2019	3	130	5	130	6.0%	0.59 [0.14, 2.52]
Izumi-2021	5	263	13	489	10.2%	0.71 [0.25, 2.01]
Monna-2021	11	168	122	2158	19.4%	1.17 [0.62, 2.21]
Guimarães-2019[API]	4	87	2	69	4.4%	1.61 [0.29, 9.09]
Strange-2020	8	180	5	217	9.0%	1.97 [0.63, 6.14]
**Subtotal (95% CI)**		**1785**		**4041**	**100.0%**	**0.70 [0.47, 1.02]**
Total events	83		247			
Heterogeneity: Tau² = 0.09; Chi² = 12.29, df = 9 (P = 0.20); I² = 27%						
Test for overall effect: Z = 1.84 (P = 0.07)						
**2.1.2 ischemic stroke**						
Durães-2016[DA]	0	15	1	12	5.5%	0.25 [0.01, 6.64]
Guimarães-2020[RIV]	3	500	7	505	32.3%	0.43 [0.11, 1.67]
Izumi-2021	4	263	9	489	42.2%	0.82 [0.25, 2.70]
Guimarães-2019[API]	4	87	2	69	20.0%	1.61 [0.29, 9.09]
**Subtotal (95% CI)**		**865**		**1075**	**100.0%**	**0.71 [0.33, 1.55]**
Total events	11		19			
Heterogeneity: Tau² = 0.00; Chi² = 1.85, df = 3 (P = 0.60); I² = 0%						
Test for overall effect: Z = 0.85 (P = 0.39)						
**2.1.4 all-cause death**						
Durães-2016[DA]	0	15	1	12	2.4%	0.25 [0.01, 6.64]
Monna-2021	10	168	342	2158	19.8%	0.34 [0.18, 0.64]
Russo-2019	1	130	2	130	4.1%	0.50 [0.04, 5.54]
Izumi-2021	11	263	24	489	18.4%	0.85 [0.41, 1.75]
Guimarães-2019[API]	7	87	6	69	12.3%	0.92 [0.29, 2.87]
Guimarães-2020[RIV]	20	500	20	505	20.1%	1.01 [0.54, 1.90]
Strange-2020	47	180	40	217	22.9%	1.56 [0.97, 2.52]
**Subtotal (95% CI)**		**1343**		**3580**	**100.0%**	**0.81 [0.47, 1.37]**
Total events	96		435			
Heterogeneity: Tau² = 0.26; Chi² = 15.35, df = 6 (P = 0.02); I² = 61%						
Test for overall effect: Z = 0.79 (P = 0.43)						
**2.1.5 cardiovascular death**						
Durães-2016[DA]	0	15	1	12	3.7%	0.25 [0.01, 6.64]
Guimarães-2019[API]	2	87	2	69	10.2%	0.79 [0.11, 5.74]
Guimarães-2020[RIV]	11	500	13	505	61.2%	0.85 [0.38, 1.92]
Izumi-2021	4	263	6	489	24.9%	1.24 [0.35, 4.45]
**Subtotal (95% CI)**		**865**		**1075**	**100.0%**	**0.89 [0.47, 1.67]**
Total events	17		22			
Heterogeneity: Tau² = 0.00; Chi² = 0.87, df = 3 (P = 0.83); I² = 0%						
Test for overall effect: Z = 0.37 (P = 0.71)						

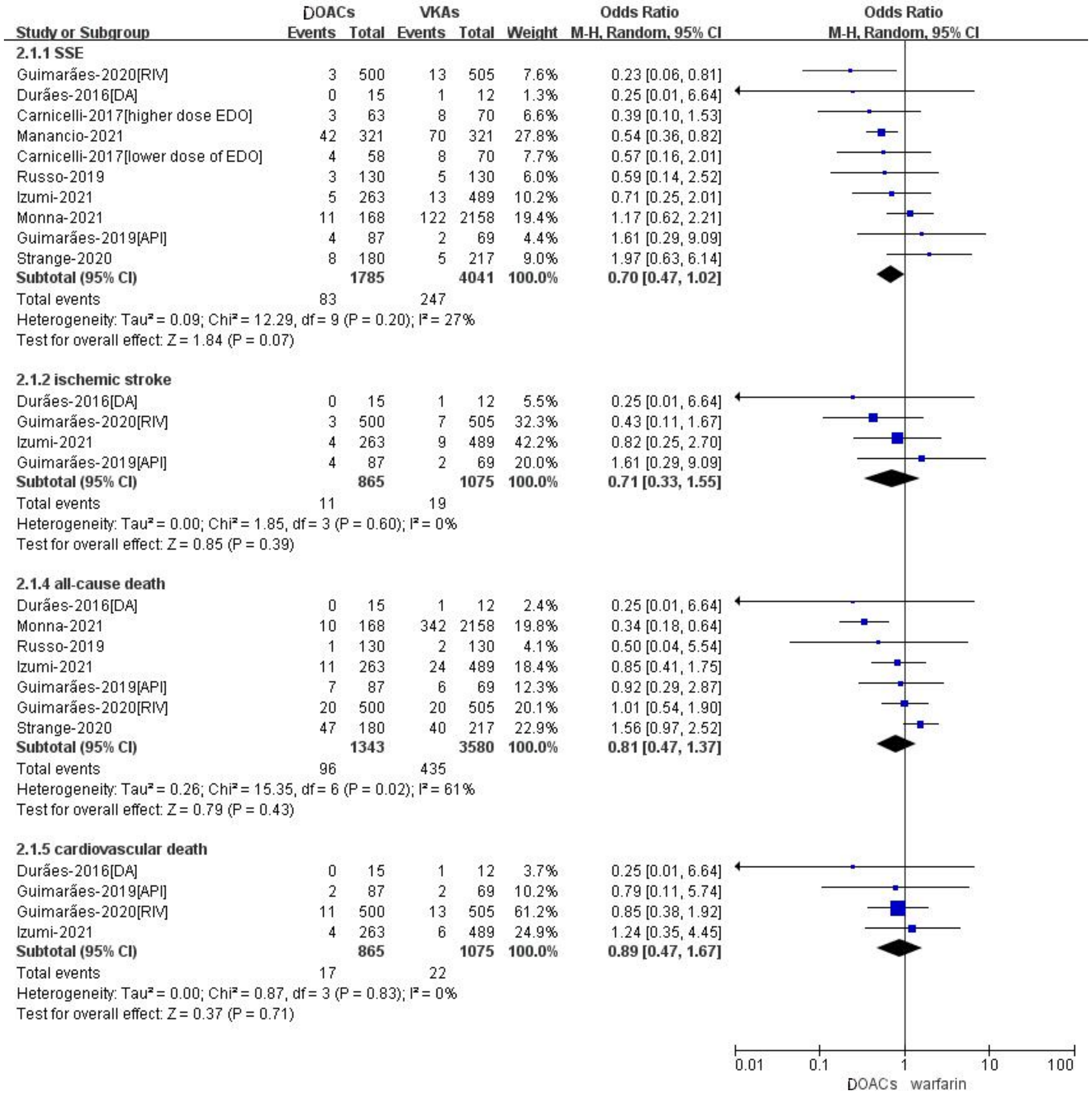

Supplementary Figure 1. Unadjusted effectiveness data of DOACs compared with VKAs among AF patients with BPHV

Study or Subgroup	DOACs Events	DOACs Total	VKAs Events	VKAs Total	Weight	Odds Ratio M-H, Random, 95% CI
**2.2.6 major bleeding**						
Carnicelli-2017[higher dose EDO]	4	63	9	70	6.3%	0.46 [0.13, 1.57]
Carnicelli-2017[lower dose of EDO]	1	58	9	70	2.5%	0.12 [0.01, 0.97]
Durães-2016[DA]	1	15	2	12	1.7%	0.36 [0.03, 4.50]
Guimarães-2019[API]	7	87	7	69	7.6%	0.78 [0.26, 2.33]
Guimarães-2020[RIV]	65	500	78	505	24.8%	0.82 [0.57, 1.17]
Izumi-2021	7	263	11	489	9.3%	1.19 [0.46, 3.10]
Manancio-2021	25	321	44	321	19.1%	0.53 [0.32, 0.89]
Monna-2021	4	168	48	2158	8.3%	1.07 [0.38, 3.01]
Russo-2019	6	130	12	130	8.6%	0.48 [0.17, 1.31]
Strange-2020	8	180	34	217	11.9%	0.25 [0.11, 0.56]
**Subtotal (95% CI)**		**1785**		**4041**	**100.0%**	**0.60 [0.42, 0.84]**
Total events	128		254			
Heterogeneity: Tau² = 0.09; Chi² = 13.71, df = 9 (P = 0.13); I² = 34%						
Test for overall effect: Z = 2.95 (P = 0.003)						
**2.2.7 any bleeding**						
Berenji-2019	3	23	3	25	4.6%	1.10 [0.20, 6.09]
Durães-2016[DA]	1	15	2	12	2.1%	0.36 [0.03, 4.50]
Guimarães-2019[API]	30	87	28	69	31.3%	0.77 [0.40, 1.48]
Guimarães-2020[RIV]	7	500	13	505	15.5%	0.54 [0.21, 1.36]
Izumi-2020	2	16	25	176	5.6%	0.86 [0.18, 4.03]
Izumi-2021	20	263	35	489	40.9%	1.07 [0.60, 1.89]
**Subtotal (95% CI)**		**904**		**1276**	**100.0%**	**0.84 [0.58, 1.21]**
Total events	63		106			
Heterogeneity: Tau² = 0.00; Chi² = 2.17, df = 5 (P = 0.83); I² = 0%						
Test for overall effect: Z = 0.95 (P = 0.34)						
**2.2.9 intracrinal bleeding**						
Guimarães-2019[API]	1	87	2	69	22.7%	0.39 [0.03, 4.39]
Izumi-2021	3	263	3	489	51.5%	1.87 [0.37, 9.33]
Russo-2019	1	130	3	130	25.7%	0.33 [0.03, 3.20]
**Subtotal (95% CI)**		**480**		**688**	**100.0%**	**0.84 [0.26, 2.66]**
Total events	5		8			
Heterogeneity: Tau² = 0.00; Chi² = 2.00, df = 2 (P = 0.37); I² = 0%						
Test for overall effect: Z = 0.30 (P = 0.76)						

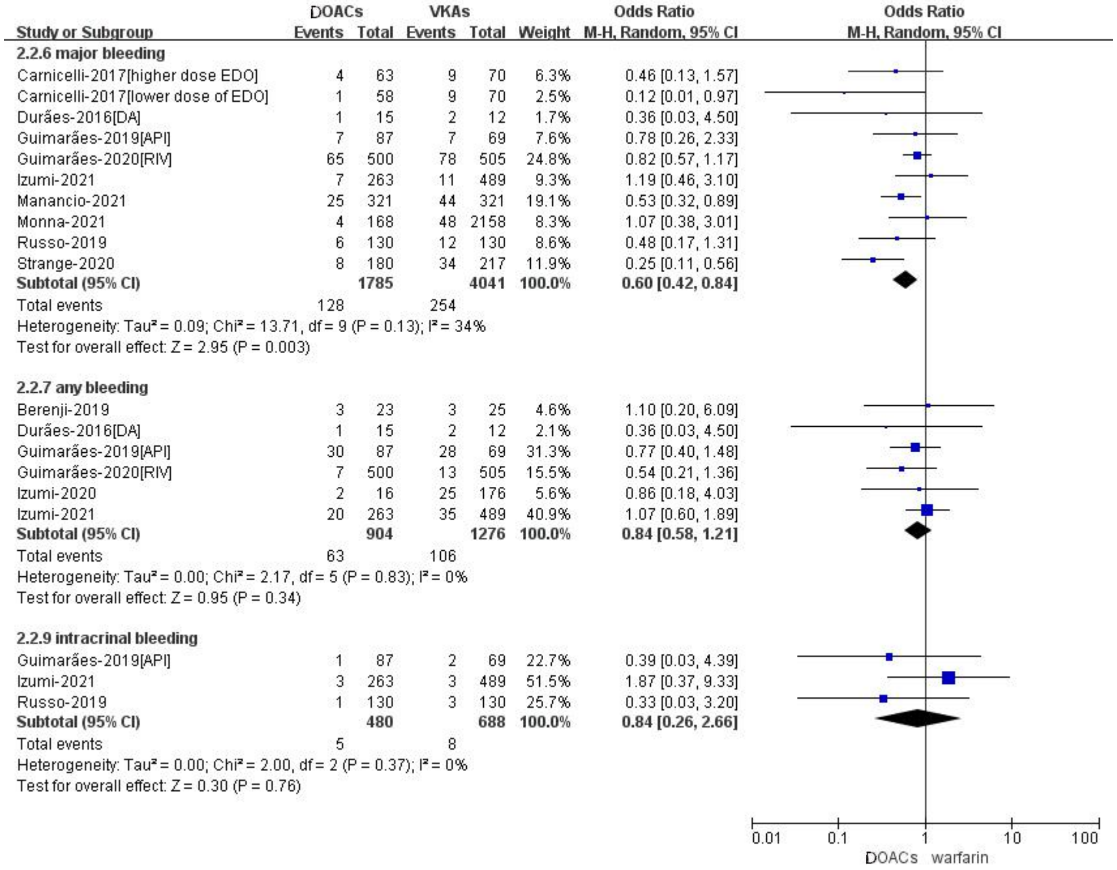

Supplementary Figure 2. Unadjusted safety data of DOACs compared with VKAs among AF patients with BPHV

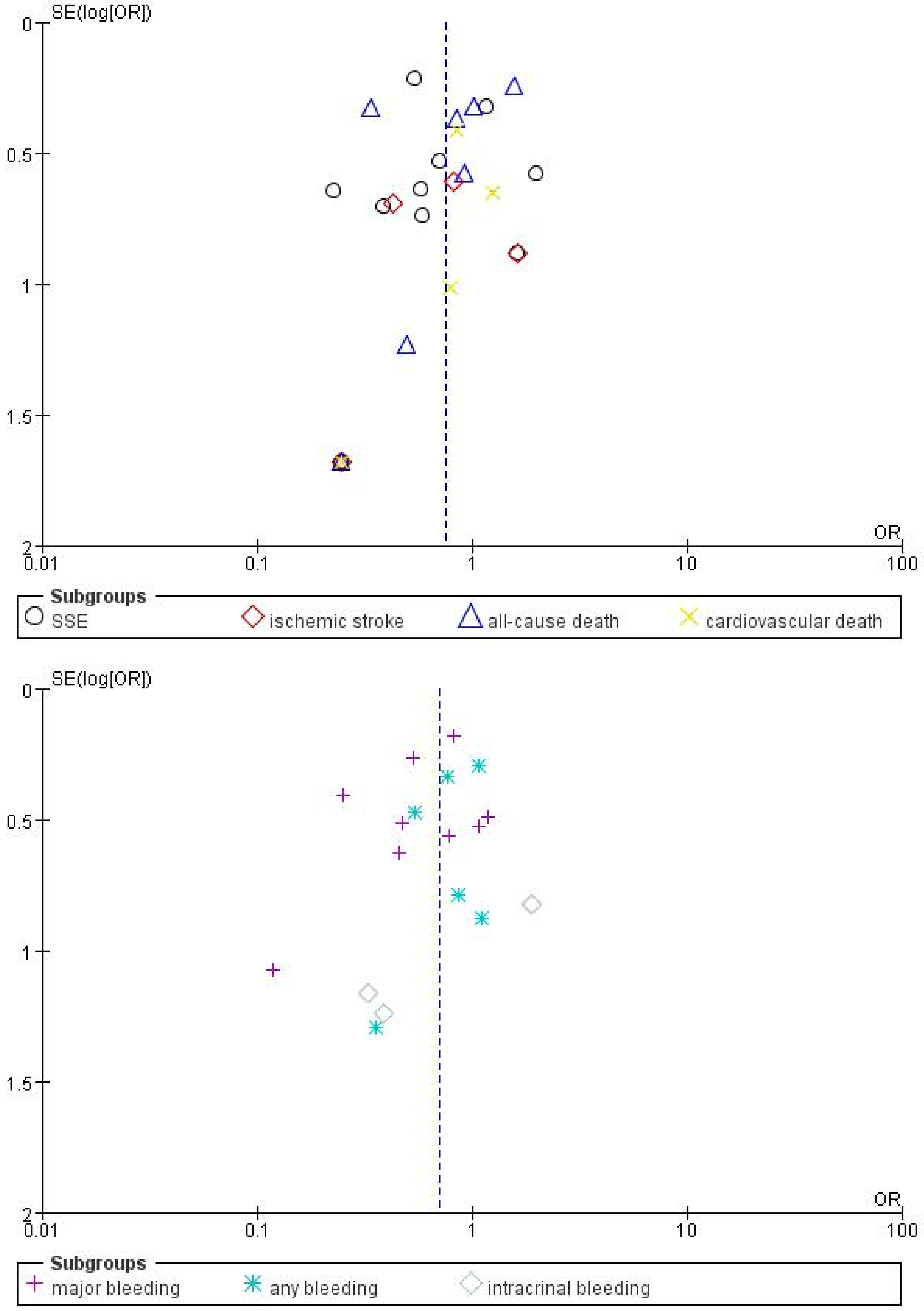

Supplementary Figure 3. The funnel plots for effectiveness and safety outcomes of DOACs versus VKAs (unadjusted data)

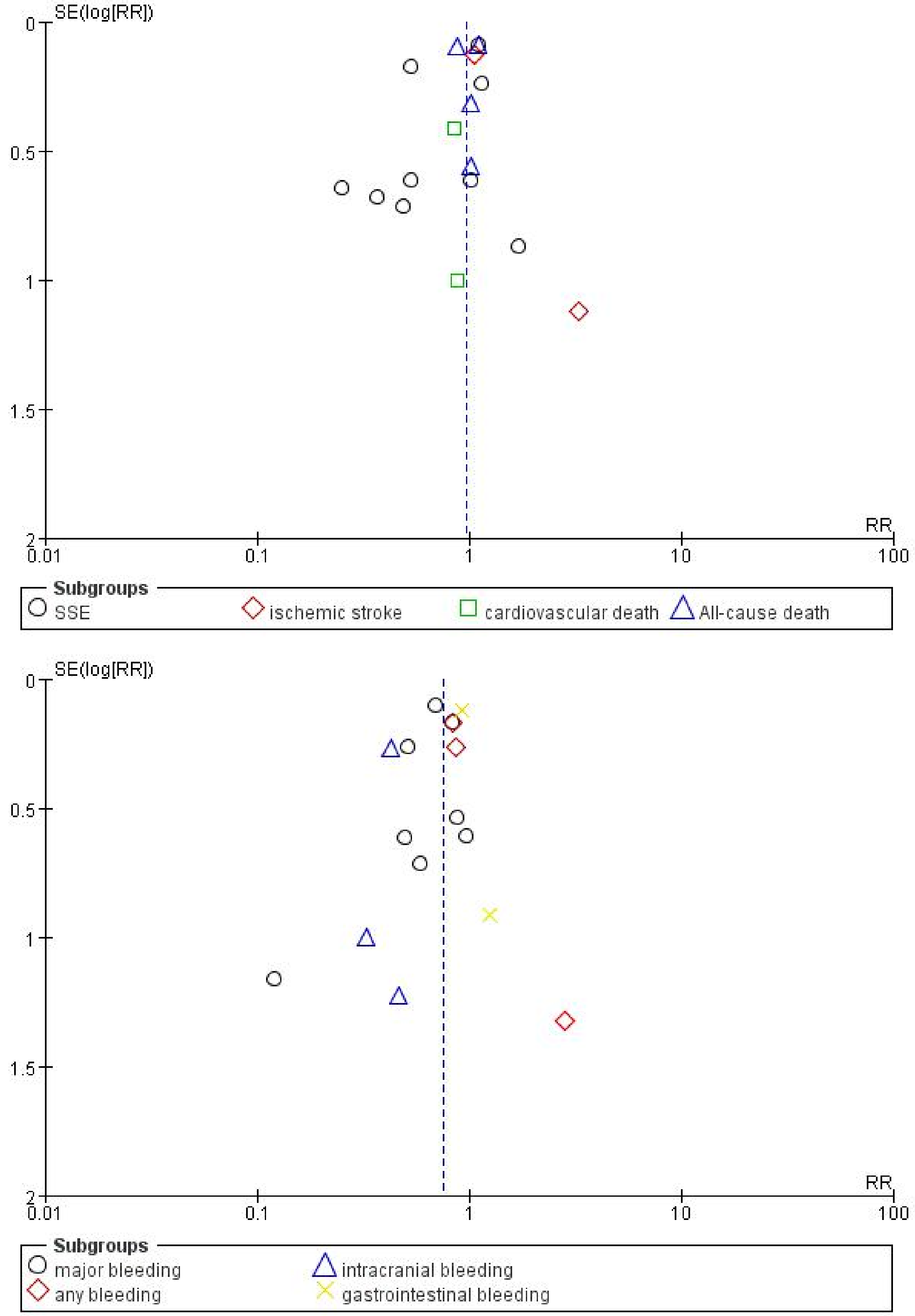

Supplementary Figure 4. The funnel plots for effectiveness and safety outcomes of DOACs versus VKAs (adjusted data)
